# Visual cortical γ−aminobutyric acid and perceptual suppression in amblyopia

**DOI:** 10.3389/fnhum.2022.949395

**Published:** 2022-09-02

**Authors:** Arjun Mukerji, Kelly N. Byrne, Eunice Yang, Dennis M. Levi, Michael A. Silver

**Affiliations:** ^1^Helen Wills Neuroscience Institute, University of California, Berkeley, Berkeley, CA, United States; ^2^Henry H. Wheeler, Jr. Brain Imaging Center, University of California, Berkeley, Berkeley, CA, United States; ^3^Vision Science Graduate Group, University of California, Berkeley, Berkeley, CA, United States; ^4^School of Optometry, University of California, Berkeley, Berkeley, CA, United States

**Keywords:** GABA, amblyopia, psychophysics, vision, inhibition, plasticity, magnetic resonance spectroscopy-MRS

## Abstract

In amblyopia, abnormal visual experience during development leads to an enduring loss of visual acuity in adulthood. Physiological studies in animal models suggest that intracortical GABAergic inhibition may mediate visual deficits in amblyopia. To better understand the relationship between visual cortical γ-aminobutyric acid (GABA) and perceptual suppression in persons with amblyopia (PWA), we employed magnetic resonance spectroscopy (MRS) to quantify GABA levels in both PWA and normally-sighted persons (NSP). In the same individuals, we obtained psychophysical measures of perceptual suppression for a variety of ocular configurations. In PWA, we found a robust negative correlation between the depth of amblyopia (the difference in visual acuity between the amblyopic and non-amblyopic eyes) and GABA concentration that was specific to visual cortex and was not observed in a sensorimotor cortical control region. Moreover, lower levels of visual cortical GABA were associated with weaker perceptual suppression of the fellow eye by the amblyopic eye and stronger suppression of the amblyopic eye by the fellow eye. Taken together, our findings provide evidence that intracortical GABAergic inhibition is an important component of the pathology of human amblyopia and suggest possible therapeutic interventions to restore vision in the amblyopic eye through enhancement of visual cortical GABAergic signaling in PWA.

## Introduction

Amblyopia is a neurodevelopmental disorder that results in deficits in multiple aspects of perception, including visual acuity, binocular vision, form vision, and motion perception (reviewed in Levi, 2013, 2020; Kiorpes, 2019). Amblyopia typically results from strabismus, anisometropia, cataract, ptosis, and/or other visual abnormalities (Webber and Wood, 2005). When these abnormalities occur during a critical period in early life, they interfere with the development of connections to and between visual cortical neurons. If amblyopia is not treated during this critical period, it can lead to perceptual deficits that persist even after correction of refractive error, ocular alignment, cataract, etc. (Fagiolini and Hensch, 2000; Levi et al., 2011; Birch, 2013). Amblyopia affects upto 3% of the world’s population (Brown et al., 2000) and is therefore of significant clinical and neuroscientific interest.

There are substantial deficits in visual perception of stimuli presented to the amblyopic eye (Hamm et al., 2014) that have been extensively characterized using established psychophysical measures (McKee et al., 2003). In addition, persons with amblyopia (PWA) typically exhibit an imbalance in interocular perceptual suppression, in which the ability of inputs representing the amblyopic eye to suppress perception in the non-amblyopic (“fellow”) eye is much weaker than suppression of perception in the amblyopic eye by stimuli presented to the fellow eye (e.g., Harrad and Hess, 1992). More recent work suggests that this asymmetry in interocular suppression contributes to many of the perceptual deficits associated with amblyopia (Li et al., 2011; Ding et al., 2013; Hess et al., 2014; Vedamurthy et al., 2015).

Physiological studies in animal models have shown that interocular suppression of visual responses occurs in early visual cortex (V1 and V2) and is correlated with the depth of amblyopia (Bi et al., 2011; Shooner et al., 2017). The primary inhibitory neurotransmitter in the brain is γ-aminobutyric acid (GABA), and visual cortical interocular suppression in animals with strabismus arises from inhibitory interactions that are mediated by GABA (Sengpiel et al., 2006). In addition, studies in human subjects with normal vision have related levels of visual cortical GABA and pharmacological manipulations of GABAergic signaling to perceptual measures of interocular suppression (Van Loon et al., 2013; Robertson et al., 2016; Mentch et al., 2019), and visual cortical GABA levels are rapidly altered by global changes in visual inputs (Lunghi et al., 2015; Kurcyus et al., 2018). GABAergic inhibition has also been linked to many aspects of visual function and development, including the onset and closure of the developmental critical period (Fagiolini et al., 2004; Sale et al., 2010).

Magnetic resonance spectroscopy (MRS) allows non-invasive measurements of GABA concentrations in awake, behaving humans (Near et al., 2013; Greenhouse et al., 2016), thereby facilitating investigation of GABA’s contributions to perception (Yoon et al., 2010; Van Loon et al., 2013). Here, we employed MRS to measure visual cortical GABA concentrations in PWA and normally-sighted persons (NSP) and correlated these GABA levels with psychophysical measures of perceptual suppression, including interocular suppression. In particular, we focused on surround suppression, in which perception of a target stimulus is impaired by simultaneous presentation of a high-contrast stimulus that surrounds or is adjacent to the target (Chubb et al., 1989; Xing and Heeger, 2001).

Results from animal studies support the idea that feedback from extrastriate visual cortical areas V2 and V3 to V1 and GABAergic inhibition are critical components of the neural circuitry that generates surround suppression (Angelucci and Bressloff, 2006; Alitto and Dan, 2010; Nassi et al., 2013). This is supported by a study in humans showing that fMRI responses in cortical area V1 to a binocularly presented stimulus are suppressed by presentation of a binocular surround (Zenger-Landolt and Heeger, 2003). In the present study, we measured GABA concentration in visual cortex using MRS and investigated its relationship to the depth of amblyopia and to psychophysical measures of surround suppression in PWA and NSP.

Both interocular and intraocular surround suppression are well-studied in NSP (e.g., Petrov and McKee, 2006; Schallmo and Murray, 2016), but there is less research investigating surround suppression in PWA. Previous work from our group has indicated increased monocular surround suppression in the amblyopic eye of PWA relative to their fellow eye and to NSP (Huh et al., 2014). Other studies have shown that presentation of a stimulus to the fellow eye results in profound perceptual suppression of a target presented to the amblyopic eye at non-overlapping retinal locations (Thompson et al., 2019), whereas presentation of a stimulus to the amblyopic eye results in weak or absent suppression of the fellow eye (Huang et al., 2012). Our study extends this work by measuring both interocular and intraocular surround suppression and correlating these psychophysical measures with visual cortical GABA concentrations in the same individuals with amblyopia.

## Materials and methods

This study consisted of both MRS and psychophysical measurements in the same individuals. Thirty-one participants completed at least one of the surround suppression psychophysical conditions. We also acquired MRS data from 15 NSP (20/20 corrected vision), 14 of whom participated in psychophysics experiments, and from 16 PWA, 8 of whom participated in psychophysics experiments. Of the 16 PWA, 12 had anisometropic amblyopia, and 4 had a mixture of anisometropia and strabismus. All participants (both NSP and PWA) were evaluated by optometry residents and students during recruitment. Visual acuity with and without correction was tested using the Bailey-Lovie LogMAR chart (Table 1), and diagnoses were confirmed, if applicable. Acuity was re-tested and confirmed immediately before the beginning of psychophysical data collection. Additional clinical details are provided in Table 1. MRS data for two subjects were discarded due to quality issues (one with severe head motion and one with inadequate visual correction), leaving MRS data for 14 PWA.

**TABLE 1:** Clinical data of persons with amblyopia (PWA).

Subject	Age	Sex	Amb type	Amb eye	Acuity in DE (logMAR)	Acuity in NDE (logMAR)
**A1**	52	F	Aniso	OD	–0.097	0.418
**A2**	30	F	Aniso	OS	–0.184	0.398
**A3**	19	M	Aniso	OD	–0.085	0.538
**A4**	50	F	Aniso	OS	–0.164	0.398
A5*	24	F	Aniso	OS	–0.057	0.281
**A6**	61	F	Aniso	OS	–0.097	0.497
A7	39	F	Aniso	OS	–0.097	0.244
A8	44	F	Aniso	OS	–0.204	0.261
A9	46	M	Aniso	OD	–0.097	0.281
A10*	33	M	Aniso	OS	–0.097	0.358
A11	36	M	Aniso	OD	–0.204	0.117
**A12**	25	M	Aniso	OS	–0.202	0.244
**M1**	43	F	Mixed	OS	–0.077	0.756
M2	33	F	Mixed	OS	–0.085	1.176
**M3**	61	F	Mixed	OS	0.077	0.602
M4	25	M	Mixed	OS	0	0.224

Bolded subjects took part in psychophysics experiments. OS, left eye; OD, right eye. *MRS data from these subjects were discarded due to quality issues (severe head motion, poor visual correction).

The PWA group was comprised of 10 female and six male participants with a mean age of 38.8 years (*SD* = 13.0). The NSP group was comprised of eight female and seven male participants with a mean age of 38.7 years (*SD* = 14.7). Subjects in the PWA group were refracted and corrected for distance vision, and all participants wore their best correction during testing.

### Magnetic resonance spectroscopy

We recorded proton MRS data using a 32-channel RF head coil in a Siemens Trio 3-Tesla MR scanner located in the Henry H. Wheeler, Jr. Brain Imaging Center. Each recording session consisted of two T1-weighted anatomical scans (sagittal MP-RAGE, TR/TE/TI = 1,900/2.52/900 ms, flip angle = 9°, FoV 250 × 176, 1 mm3 voxel size, acceleration factor of two) and eight MEGA-PRESS scans [320 transients per scan − 160 Off and 160 On, TR/TE = 1,500/68 ms, edit pulse frequencies of 1.9 ppm (On-resonance) and 7.5 ppm (Off-resonance), edit pulse bandwidth of 45 Hz, delta frequency of −1.7 ppm relative to water (chosen for signal detection at 3.00 ppm), water suppression bandwidth of 50 Hz, TA = 8.4 min]. MEGA-PRESS scans were collected in pairs, switching between On- and Off-resonance editing pulses.

**FIGURE 1:**
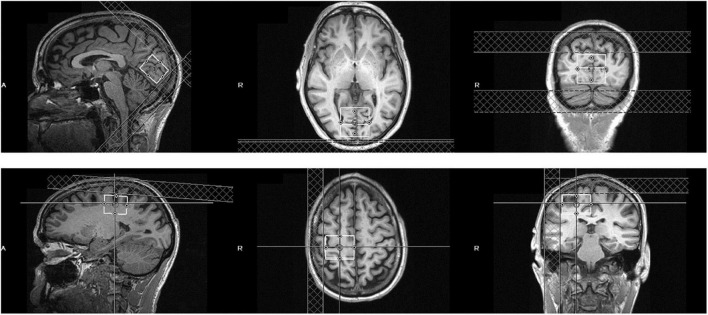
Position of the visual cortical MRS voxel **(top row)** and sensorimotor control voxel **(bottom row)** in an example subject. The voxel is 3 × 3 × 3 cm and centered bilaterally over the calcarine sulcus (visual cortex) or right precentral gyrus (sensorimotor control).

MRS data were acquired from a 3 × 3 × 3 cm occipital cortical voxel centered bilaterally on the calcarine sulcus and parallel to the parieto-occipital sulcus (Figure 1). Another 3 × 3 × 3 cm voxel served as a control and was centered over the hand knob area of the precentral gyrus in the right hemisphere. This control sensorimotor voxel was parallel to the superior-posterior axis, with the medial border of the voxel abutting the longitudinal fissure. This sensorimotor cortical area is easily defined based on gross anatomical landmarks and has been used as a region of interest in multiple previous MRS studies (Evans et al., 2010; Stagg et al., 2011; Greenhouse et al., 2016). Participants were asked to maintain fixation on a central point either on a uniform gray background or a contrast-reversing checkerboard visual stimulus that was presented to one or both eyes. GABA MRS measurements were averaged across these visual stimulation conditions. This procedure was based on results from MRS studies at high magnetic field strength that found no significant changes to visual cortical GABA levels due to visual stimulation (Lin et al., 2012; Schaller et al., 2013).

Spectroscopy data were analyzed according to the procedure described in Greenhouse et al. (2016). Sets of 10 consecutive transients were averaged and stored in a single Siemens rda file. This resulted in 32 rda files per scan (16 On and 16 Off). The data were preprocessed and analyzed with custom Matlab code implemented by Greenhouse et al. (2016). Analysis code can be downloaded at.^1^ Preprocessing included zero-padding from 1,024 to 4,096 data points and apodization with a 4-Hz Gaussian function. Off-resonance spectra were manually phase-corrected and aligned using creatine (Cr) as a reference. Correction values were applied to the paired On-resonance spectra (Evans et al., 2013; Near et al., 2014). Summary statistics were calculated for each frequency of each On- and Off-resonance spectrum, and the number of deviant values (>2 standard deviations from the mean) was tallied. The spectra were also visually inspected, and 7.7% of total spectra were excluded from further analysis, based on the number of deviant values and overt corruption or distortion of the spectra (Near et al., 2013; Simpson et al., 2017). Metabolite concentrations were estimated using peak integration methods that have been applied by others (Yoon et al., 2010; Greenhouse et al., 2016; Maddock et al., 2016). GABA concentrations were calculated from the signal range of 2.85 and 3.15 ppm in the difference spectra, and creatine concentrations were calculated from the signal range of 2.93 and 3.10 ppm in the summed On- and Off-resonance spectra. GABA concentrations were then normalized by creatine by calculating the ratio of total GABA/total Creatine for each scan.

We found no significant difference between PWA and NSP in the proportion of gray matter in either the visual cortical or sensorimotor control MRS voxels [visual: *t*_(27)_ = 0.81, *p* = 0.42; motor: *t*_(27)_ = 0.96, *p* = 0.34]. Given this result, we did not perform additional normalization of the GABA:creatine ratio based on the proportion of gray matter.

### Psychophysics

#### Apparatus

Stimuli were generated with MATLAB and the Psychophysics Toolbox (Brainard, 1997; Pelli, 1997) and were presented on the left and right halves of a gamma-corrected CRT monitor. We used a 1920 × 1440 screen resolution and a 75 Hz refresh rate. Stimuli were viewed centrally through a mirror stereoscope at a distance of 60 cm in a darkened room. Stimuli were always presented on a uniform gray background at mean luminance.

#### Stimuli and experimental procedures

Surround suppression is stronger for peripheral targets compared to foveal targets (Snowden and Hammett, 1998; Petrov et al., 2005; Lev and Polat, 2011). Thus, we chose to measure surround suppression in the near periphery with an annulus-shaped stimulus similar to those used in previous studies by our group as well as others (Zenger-Landolt and Heeger, 2003; Yoon et al., 2010; Kosovicheva et al., 2012). In addition, surround suppression is more pronounced for iso-oriented compared to cross-oriented surrounds (Xing and Heeger, 2001; Yoon et al., 2009). We therefore also measured the orientation selectivity of surround suppression and its relationship to visual cortical GABA.

**FIGURE 2:**
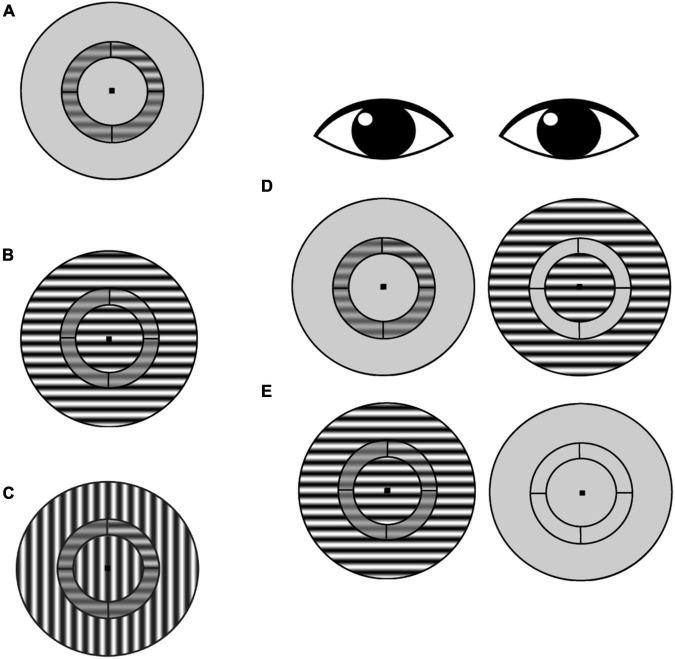
Surround suppression task. **(A)** Subjects were asked to identify which annulus quadrant contained a contrast increment relative to the pedestal contrast (in this example, the target contrast increment is in the upper right quadrant). This no-surround condition was used to estimate baseline contrast discrimination thresholds for each participant. **(B)** Iso-oriented condition: target-containing annulus and surround share the same orientation. **(C)** Cross-oriented condition: annulus and surround have orthogonal orientations. **(D)** Schematic of dichoptic presentation. The annulus was presented to one eye, and the surround was presented to the other eye. In this example, the surround is iso-oriented [as in **(B**)], but cross-oriented surrounds [as in **(C)**] were also tested. **(E)** Schematic of monocular presentation, with annulus and surround presented to one eye and only the fixation point presented to the other eye.

The stimulus was a contrast reversing (4 Hz), sine-wave grating (1 cpd spatial frequency) presented within a circular aperture (Figure 2). Concentric black lines divided the stimulus into an annulus (in which the target was presented) and inner and outer surround regions. The annulus extended 3° to 4.5° (radii) of eccentricity from the center of the stimulus, and the outer surround region had a maximum eccentricity of 8° radius. The grating orientation within the annulus was always horizontal. In the iso-oriented condition, the inner and outer surround regions consisted of horizontal gratings that were phase aligned with the gratings in the annulus, and in the cross-surround condition, the gratings within the surround regions were vertically oriented. A black fixation point was present at the center of the image for both eyes at all times. Participants viewed the stimuli through a custom mirror stereoscope, and correct alignment of the two eyes’ images was achieved by adjusting the mirrors so that vertical and horizontal Nonius lines could be seen by each eye and appeared as a cross. In addition, binocular fusion contours (seen by both eyes) encircling the stimuli were present at all times to promote stable binocular eye alignment.

The annulus was divided by black lines into four quadrants of equal (30% Michelson) contrast. Participants were asked to indicate *via* keypress which quadrant contained the contrast increment (4-alternative forced choice). On each trial, the annulus and surround regions were simultaneously presented for 1 s to either the same eye (monocular trials) or to the two eyes separately (dichoptic trials). For baseline trials (no surround), the annulus was presented to one eye, while the other eye viewed the mean luminance background. The amount of contrast increment was determined for each trial using a 2-up, 1-down staircase procedure in steps of 0.125 log units. Each staircase terminated after 12 reversals, and contrast discrimination threshold for each staircase was defined as the average of the last six reversals. Participants had unlimited response time after stimuli were removed from the screen, and they were provided with auditory feedback after every trial. Participants completed practice trials at the beginning of every session.

Surround regions were presented at 3–5 different Michelson contrast values that were individually determined for each participant. During the pilot phase of this study, we observed that some PWA failed to perceive even a maximum-contrast (99% Michelson) annulus with their amblyopic eye when the maximum-contrast surround was presented to the non-amblyopic eye. As a result, they were unable to detect any contrast increment within the annulus with their amblyopic eye. We therefore determined the maximum surround contrast at which thresholds could be estimated in all presentation conditions prior to the start of data collection for each participant. This contrast was then used as the maximum of the range of tested surround contrasts for a given participant, and the set of tested surround contrasts was identical across all experimental conditions for that participant.

### Experimental design and statistical analysis

#### Presentation conditions

Participants were tested in eight different experimental conditions in a 2 (eye) × 2 (ocular configuration of center and surround stimuli; monocular or dichoptic) × 2 (relative surround orientation; cross- or iso-) factorial design plus a baseline (no surround) condition for each eye (Table 2). Trials in a given experimental condition were blocked, and within each block, staircases with different surround contrast values were randomly interleaved. For each participant, there were four staircases per experimental condition per surround contrast value. Data from additional staircases were collected when preliminary threshold estimates (computed as the average of the last six reversals) failed to converge.

**TABLE 2:** Schematic of surround suppression presentation conditions.

Condition	Monocular DE		Monocular NDE		Dichoptic DE		Dichoptic NDE	
Eye	Annulus	Surround	Annulus	Surround	Annulus	Surround	Annulus	Surround
DE	✓	✓	–	–	✓	–	–	✓
NDE	–	–	✓	✓	–	✓	✓	–

DE, dominant eye (fellow eye for PWA). NDE, non-dominant eye (amblyopic eye for PWA).

For PWA, the amblyopic eye was the non-dominant eye (NDE), and the fellow eye was the dominant eye (DE). For NSP, the NDE was defined as the eye with the higher baseline (without a surround) contrast discrimination threshold, and the other eye was the DE. This definition is similar to others that have been used to measure sensory eye dominance (e.g., Wang et al., 2018), and it is more relevant to our study of perceptual measures than methods based on sighting dominance (Mapp et al., 2003).

In the monocular conditions, the annulus and surround were presented to the same eye (either monocular-DE or monocular-NDE) (Table 2). When the annulus and surround were shown to different eyes (dichoptic conditions), the conditions were labeled according to which eye viewed the annulus. Thus, in the dichoptic-DE condition, the annulus was presented to the dominant eye and the surround to the non-dominant eye. The reverse ocular configuration was used in the dichoptic-NDE condition (Table 2). Taken together, these conditions allowed measurement of both directions of interocular surround suppression (DE suppressing NDE and NDE suppressing DE) as well as monocular surround suppression in both the DE and NDE.

#### Threshold estimation

**FIGURE 3:**
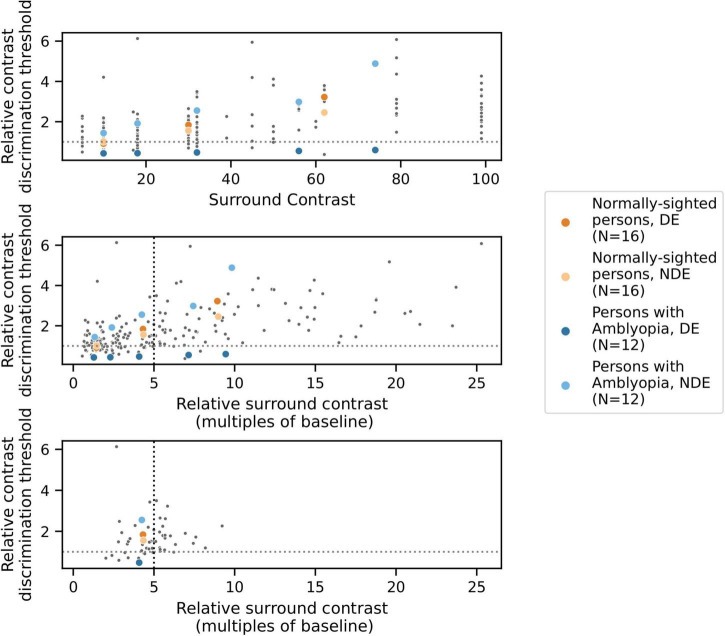
Results from one example condition (iso-oriented surround, dichoptic presentation) illustrating the psychophysical modeling approach. Data points indicate relative contrast discrimination thresholds for each eye when the surround was presented to the other eye. Observations from one PWA (blue) and one NSP (orange) are shown in color. **Top:** Normalized contrast discrimination threshold (y-axis) vs. unnormalized surround contrast (x-axis, expressed as Michelson contrast values). The y-axis indicates contrast discrimination thresholds that were normalized by the baseline (no surround) contrast discrimination threshold for each eye and each participant. As a result of the normalization, each subject’s baseline values were rescaled to 1 for each eye and condition, and the horizontal line at y = 1 separates surround suppression (>1) from facilitation (<1). **Middle:** After normalization of surround contrasts. The x-axis represents the contrast of the surround as a multiple of the same baseline contrast discrimination threshold value used to normalize the contrast discrimination threshold values. The black vertical dotted line indicates the relative surround contrast value of five that we used to calculate a single relative contrast discrimination threshold value for each combination of eye, condition, and participant. Each of these thresholds was derived based on the relative surround contrast value that was closest to five. **Bottom:** The selected data points that were used for further analyses.

For each condition and participant, we estimated the contrast increment needed to reliably identify the target quadrant (defined as the average of the contrast increment values of the last six reversals of each psychophysical staircase). Contrast discrimination thresholds were estimated for two baseline conditions in which a surround was absent (one for each eye) and for the eight conditions with a surround shown in Table 2, each tested with 3–5 different surround contrast values. These values ranged from 5 to 99% Michelson contrast (full data in Figure 3, top panel).

#### Psychophysical modeling

To correct for differences in baseline contrast discrimination thresholds across subjects and eyes, normalized values of both the estimated threshold and the presented surround contrast values were calculated by dividing by the subject’s baseline (no surround) threshold for the eye which viewed the annulus, generating “relative threshold” and “relative surround contrast” values that were used for further analyses. As a result, a different set of relative surround contrast values were associated with each eye of each participant. However, to conduct group comparisons and correlations with visual cortical GABA levels, it is useful to obtain a single measure of contrast discrimination for each combination of eye, condition, and participant. We therefore initially attempted to fit the relative threshold vs. relative surround contrast function for each condition (Figure 3) with a model in order to derive a relative threshold level for specified relative surround contrast values, but there were often conditions that were not well fit by the model.

We therefore used a data-driven approach to obtain a single relative surround contrast level for the entire data set that was then used to compute a contrast discrimination threshold value for each combination of eye, condition, and participant. Specifically, we determined the relative surround contrast value across all of the data combined across all participants that satisfied the following constraints: (1) reliably evoked surround suppression (mean relative threshold > 1, indicating higher thresholds in the presence of a surround), and (2) unbiased, in that analysis of data from one eye was not systematically based on higher relative surround contrasts compared to data from the other eye. Note that meeting the second criterion requires normalization by each eye’s baseline contrast threshold, since the two eyes of PWA differ in absolute contrast sensitivity. In our data set, these constraints were best satisfied by a relative surround contrast value of approximately five (i.e., five times the baseline contrast discrimination threshold). For further analysis of each combination of eye, condition, and participant, we used the relative contrast discrimination threshold value from the data point that was closest to a relative surround contrast value of five (Figure 3). We also conducted our analyses over a range of relative surround contrast values from 2 to 10, and all results that are reported as statistically significant in this paper were robust across this range.

#### Statistics and tests

For all correlation analyses, we employed the Spearman’s correlation coefficient (ρ) as a non-parametric measure of the strength of the relationship. Unlike the Pearson’s correlation coefficient r, Spearman’s ρ is calculated on ranks and therefore can reveal both linear and non-linear correlations. Like Pearson’s r, it ranges from -1 (indicating perfect negative correlation) to + 1 (perfect positive correlation).

In addition, we used permutation testing to generate 100,000-element distributions of ρ values under the null hypothesis, thereby avoiding assumptions about the shapes of the distributions of the recorded data. For each statistical test involving correlations, we rejected the null hypothesis if the observed ρ was more extreme than 95% of the values in the null distribution, corresponding to a two-tailed test with α = 0.05.

To characterize the relationships between visual cortical GABA and perceptual surround suppression, we first tested whether the Spearman’s correlation coefficient ρ was significantly different from 0 for each eye and ocular configuration, indicating evidence for an association between GABA and the magnitude of surround suppression for that eye and ocular configuration. Secondly, we compared ρ values between the two eyes for the two complementary dichoptic presentations, allowing us to test for possible interocular differences in the relationship between GABA and surround suppression. Analogous tests were conducted for the monocular conditions. Finally, we compared the amplitude of interocular differences in surround suppression between PWA and NSP to test for differences between groups.

## Results

**FIGURE 4:**
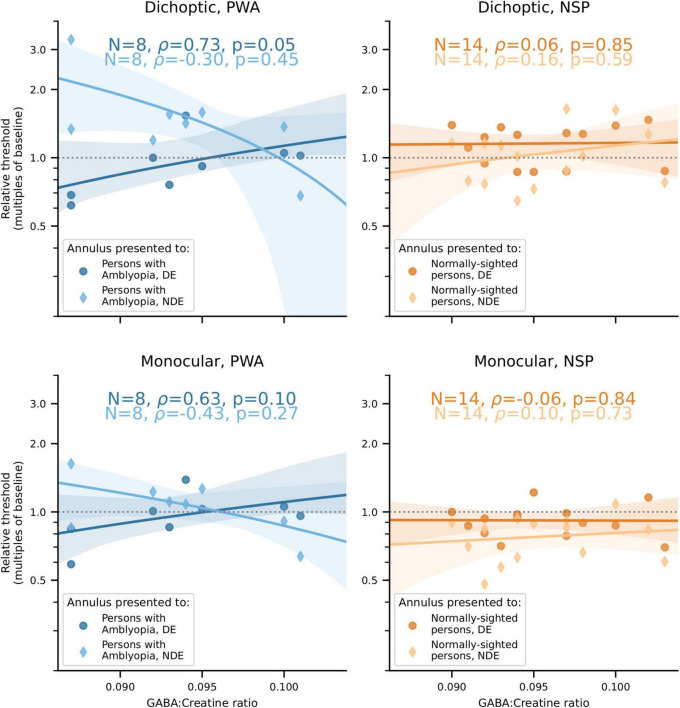
Relationships between cross-oriented surround suppression (Figure 2C) and visual cortical GABA levels. **Top row:** Dichoptic presentation. **Bottom row:** Monocular presentation. **Left column:** PWA. **Right column:** NSP. Shading indicates 95% bootstrapped confidence intervals of the best linear fit. The difference in the strength of the GABA/surround suppression correlation between the two eyes of PWA was significant for monocular (*p* = 0.04) and dichoptic (*p* = 0.048) presentation.

**FIGURE 5:**
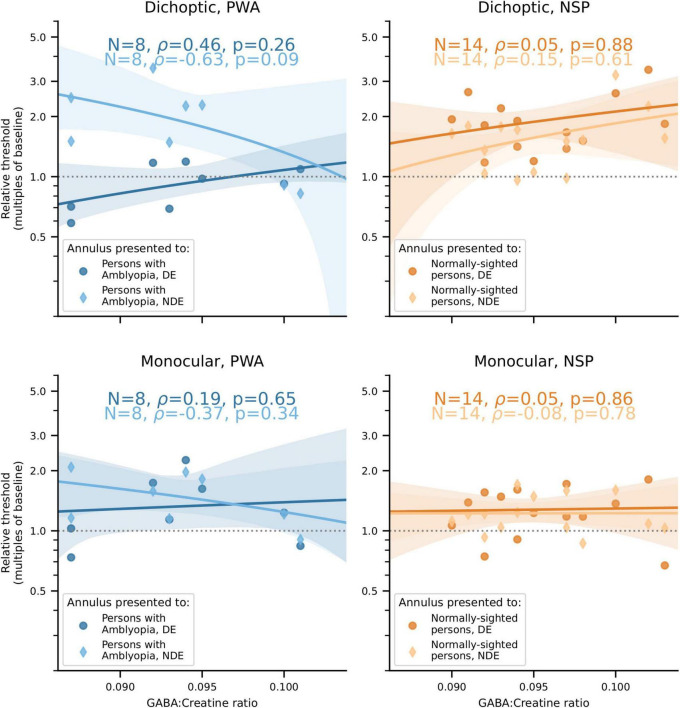
Relationships between iso-oriented surround suppression (Figure 2B) and visual cortical GABA levels. **Top row:** Dichoptic presentation. **Bottom row:** Monocular presentation. **Left column:** PWA. **Right column:** NSP. Shading indicates 95% bootstrapped confidence intervals of the best linear fit. The difference in the strength of the GABA/surround suppression correlation between the two eyes of PWA was significant for dichoptic presentation (*p* = 0.036).

Our main finding was that the correlations between visual cortical GABA and interocular perceptual suppression are different for the two eyes of PWA. This difference was due to a more positive correlation of visual cortical GABA with suppression of the dominant eye (DE) and a more negative correlation of GABA with suppression of the non-dominant eye (NDE). This pattern was observed for both cross-oriented surrounds (Figure 4) and iso-oriented surrounds (Figure 5). For cross-oriented surrounds, this difference between the DE and NDE was significant for both monocular (*p* = 0.04) and dichoptic (*p* = 0.048) presentation conditions. For iso-oriented surrounds, this difference was significant for dichoptic presentation (*p* = 0.036).

When these analyses were carried out using GABA concentrations from the sensorimotor control voxel, none of these correlations or differences were significant (all *p* > 0.18 except one *p* = 0.10). Additionally, we tested for anatomical specificity by comparing the difference of correlation coefficient values obtained from the visual cortical and from the sensorimotor control MRS voxels to the permutation distribution of these difference values generated by the bootstrap procedure. While these tests were not significant for any measure (all *p* > 0.07), several approached significance with *p* < 0.10. The consistent and significant results observed with visual cortical (but not sensorimotor) GABA concentrations provides some support for the finding that correlations between visual cortical GABA and interocular perceptual suppression are different for the two eyes of PWA.

In the following sections we show how we arrived at these conclusions, beginning with presentation of MRS results, then psychophysical results, and finally correlations between these brain and behavioral measures.

### No significant difference between persons with amblyopia and normally-sighted persons in visual cortical γ-aminobutyric acid levels

**FIGURE 6:**
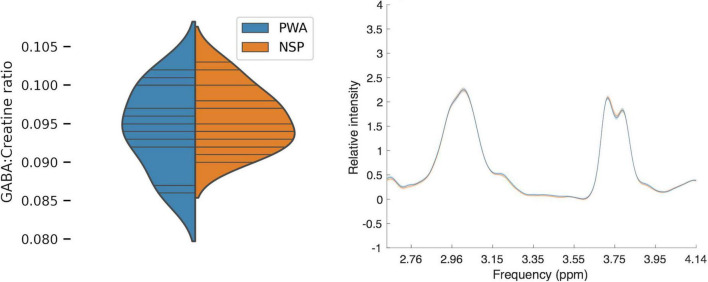
**Left:** Plot of MRS GABA:creatine ratio values in visual cortex. Blue, persons with amblyopia (*N* = 14; mean = 0.09, SEM = 0.001). Orange, normally sighted persons (*N* = 15; mean = 0.09, SEM = 0.001). Horizontal lines indicate visual cortical GABA levels in individual participants, and plotted distributions are kernel density estimates. There was no significant difference in visual cortical GABA levels between the two groups. **Right:** Mean normalized MRS spectra for PWA (blue) and NSP (orange). Width of colored regions indicates standard error of the mean. The mean normalized spectra are very similar for the two groups.

We first compared visual cortical GABA levels for PWA and NSP by using the ratio of GABA to creatine within the MRS voxel for each subject (Figure 6). We did not find a significant difference in visual cortical GABA levels between PWA and NSP [two-tailed *t*-test; *t*_(27)_ = −0.75; *p* = 0.46].

### Depth of amblyopia is significantly inversely correlated with visual cortical γ-aminobutyric acid in persons with amblyopia

**FIGURE 7:**
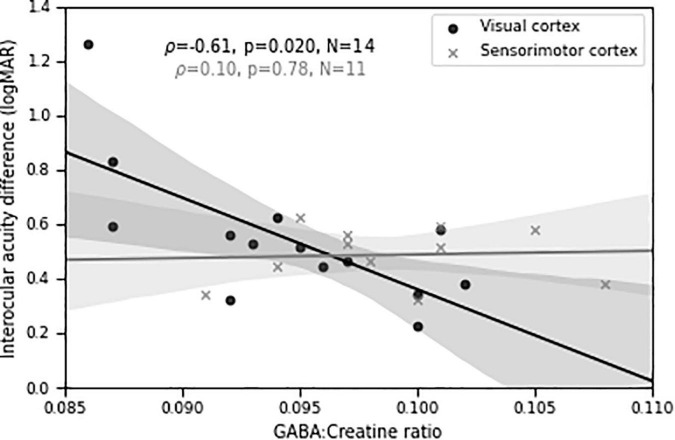
Visual cortical GABA levels predict differences in visual acuity between the two eyes in persons with amblyopia. Shaded regions are bootstrapped 95% confidence intervals of the best linear fit.

Importantly, we found a significant negative relationship (ρ = −0.61, *p* = 0.02, *N* = 14) between visual cortical GABA concentration and depth of amblyopia, as measured by interocular difference in visual acuity (Figure 7). That is, lower levels of GABA were associated with more severe amblyopia. A control MRS voxel located in sensorimotor cortex showed no significant relationship (ρ = 0.10, *p* = 0.78, *N* = 11). The difference in Fisher-transformed correlation values between GABA MRS levels in the two cortical locations corresponds to a z-score of 1.74 and a two-tailed *p*-value of 0.08. This result, combined with our findings of different correlations between perceptual suppression and visual cortical GABA for the DE and NDE in PWA (Figures 4, 5), provide further evidence of a relationship between abnormal interocular interactions and reduced GABAergic visual cortical inhibition in amblyopia.

### Interocular differences in contrast discrimination in persons with amblyopia

**FIGURE 8:**
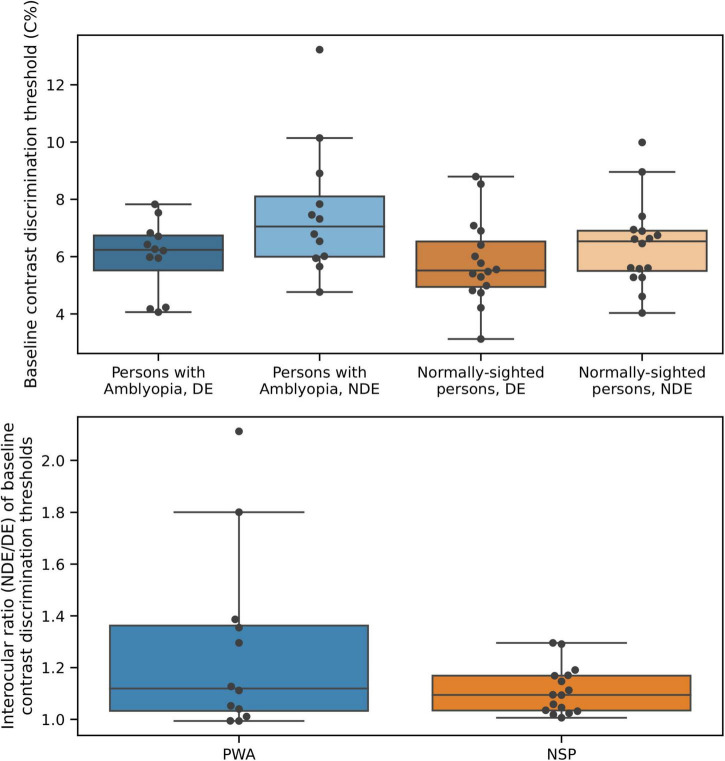
Baseline contrast discrimination thresholds differ between the two eyes of persons with amblyopia. **Top:** Baseline contrast discrimination thresholds. **Bottom:** Interocular difference in baseline contrast discrimination thresholds.

Baseline (i.e., in the absence of a surround) contrast discrimination thresholds are plotted in Figure 8. As expected from the literature on perceptual deficits in amblyopia (e.g., Levi, 2013), contrast discrimination thresholds were lower in the fellow (dominant, DE) eye of PWA compared to the amblyopic (non-dominant, NDE) eye [two-tailed *t*-test; *t*_(11)_ = 2.68; *p* = 0.02]. The NSP group did not have a clinically designated dominant eye, so the eye with the lower baseline contrast discrimination threshold was defined as the DE and the eye with the higher threshold as the NDE for participants in this group. We do not present results from inferential statistical testing of interocular differences in contrast discrimination threshold values for NSP, as the classification of the DE and NDE was based on these threshold values for this group. The effect size was smaller for PWA (0.77) than for NSP (1.21).

### Interocular perceptual suppression of the non-dominant eye by the dominant eye is significantly greater than that of the dominant eye by the non-dominant eye in persons with amblyopia

An omnibus ANOVA (Table 3) of the surround suppression data showed significant main effects of surround orientation (cross- or iso-oriented surround), ocular configuration (dichoptic or monocular), and eye (DE or NDE). The main effect of group (PWA or NSP) was not significant. In addition, three significant interactions were observed: Ocular Configuration × Eye, Group × Eye, and Ocular Configuration × Group × Eye.

**TABLE 3:** Omnibus ANOVA results.

Effect	*F*	*p* (>F)
Orientation	34.41	1.74 × 10^–8^*
Ocular configuration	19.55	1.58 × 10^–5^*
Eye	6.94	0.009*
Group	2.26	0.134
Ocular configuration × eye	4.80	0.030*
Group × eye	24.87	1.29 × 10^–6^*
Ocular configuration × group × eye	9.46	2.38 × 10^–3^*

The symbol * indicates the results that are significant at a p < 0.05 level.

**FIGURE 9:**
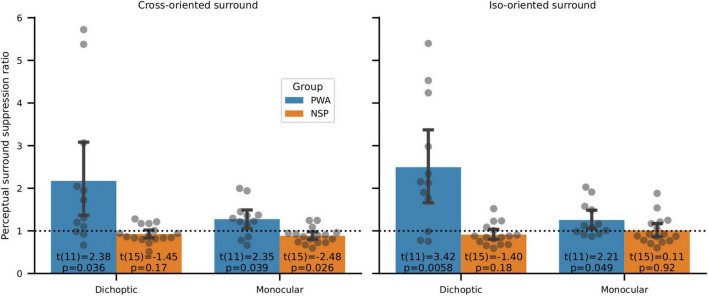
Perceptual surround suppression. Data points are NDE:DE suppression ratios for individual subjects. Bars indicate means and standard errors. In PWA, one-sample *t*-tests of whether suppression ratios were different from a value of 1 showed significantly more suppression of the DE than the NDE in all four conditions (cross- and iso-oriented surrounds and dichoptic and monocular conditions). Suppression ratios were not significantly different from a value of 1 for NSP in 3 out of 4 conditions, and the fourth condition (cross-oriented surround, monocular presentation) showed a significant difference in the opposite direction from PWA (i.e., more suppression in the NDE than the DE).

When the annulus and surround were presented to different eyes (dichoptic presentation) in PWA, we found significantly more interocular perceptual suppression of the NDE by the DE compared to the amount of suppression of the DE by the NDE (Figure 9). In particular, we used two-tailed one-sample *t*-tests to evaluate whether the ratio of suppression for annuli presented to the NDE vs. to the DE was different from a value of 1. This ratio was unequal and highly significant for both cross-oriented and iso-oriented surrounds in PWA. No such significant differences were observed for NSP for dichoptic presentation. Note that none of these differences in PWA are due to interocular differences in contrast discrimination, as surround suppression values were calculated from data that were normalized by contrast discrimination threshold values obtained from the baseline (no surround) condition for each eye of each participant (Figure 3).

When the annulus and surround were presented to the same eye (monocular condition), we observed greater suppression in the NDE than the DE in PWA (Figure 9). This difference was significant for both cross-oriented and iso-oriented surrounds. We observed the opposite pattern in the NSP group (more suppression in the DE than the NDE), and this difference was significant for monocular presentation.

For PWA, stereo vision was assessed with the Randot Circles test during clinical assessment. Stereoacuity was not significantly correlated with interocular perceptual suppression ratio for any of the four conditions (cross- and iso-oriented surrounds, dichoptic and monocular conditions; all |ρ| < 0.44, all *p* > 0.15) or with the depth of amblyopia (ρ = 0.26, *p* > 0.37). Additionally, depth of amblyopia was not significant correlated with interocular perceptual suppression ratio for any of the four conditions (all | ρ| < 0.27, all *p* > 0.53).

### Replication of previous reports of psychophysical orientation-selective surround suppression

**FIGURE 10:**
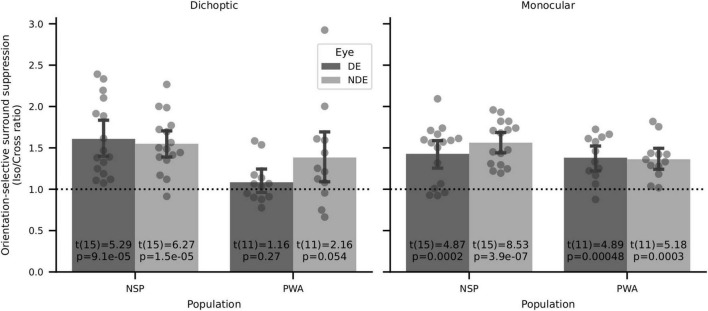
Orientation-selective surround suppression (OSSS) results. Two-sided one-sample *t*-tests of iso/cross suppression ratios showed significant OSSS in 6 of 8 conditions, including all four for normally sighted persons.

Surround suppression has previously been shown to be orientation-selective, with iso-oriented surrounds producing stronger perceptual suppression than cross-oriented surrounds (Xing and Heeger, 2001; Yoon et al., 2009). We tested whether these previous findings of perceptual orientation-selective surround suppression (OSSS) were replicated in our data set. We calculated the orientation selectivity of surround suppression for each participant by computing the ratio of the relative contrast discrimination threshold values in the iso- and cross-oriented surround conditions. We then used two-tailed one-sample *t*-tests to evaluate whether these interocular OSSS ratios were different from a value of 1. We found strong evidence of orientation-selective surround suppression for six out of eight configurations that we tested (Figure 10). This result is in agreement with prior literature on orientation-selective surround suppression (Xing and Heeger, 2001; Yoon et al., 2009). The only conditions in which highly significant OSSS was not observed were in PWA with dichoptic presentation, where interocular differences due to amblyopia may have overwhelmed detection of OSSS.

### No significant correlation between visual cortical γ-aminobutyric acid and the strength of orientation-selective surround suppression

We also analyzed the relationship between visual cortical GABA and orientation-selective surround suppression (the ratio of relative contrast discrimination threshold values for iso- and cross-oriented configurations). We found strong orientation-selective surround suppression when considering all participants who took part in the psychophysics experiments (Figure 10), and this was also evident in the subset of subjects who had both MRS and psychophysical data (14 NSP and 8 PWA). However, in contrast to the results reported in Yoon et al. (2010), we observed no significant correlations between the strength of this orientation-selective surround suppression and visual cortical GABA levels (all |ρ| < 0.5, all *p* > 0.2).

### No significant relationship between visual cortical γ-aminobutyric acid and contrast discrimination in either persons with amblyopia or normally-sighted persons

**FIGURE 11:**
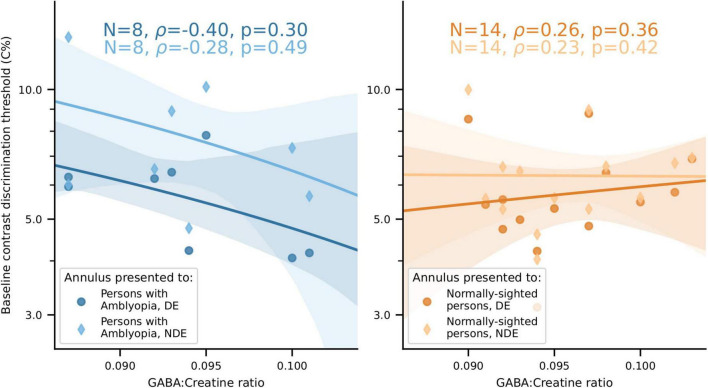
No significant relationship between visual cortical GABA levels and baseline contrast discrimination thresholds for either PWA or NSP. Left, PWA. Right, NSP. Marker shape and color indicate the eye that viewed the annulus. Shaded regions are bootstrapped 95% confidence intervals of the best linear fit.

We found no significant relationship between visual cortical GABA levels and baseline (no-surround) contrast discrimination in either PWA or NSP (Figure 11).

## Discussion

We found that, on average, PWA do not have significantly different levels of visual cortical GABA compared to NSP. However, we observed a significant negative correlation between visual cortical GABA concentration and depth of amblyopia in this group, as measured by interocular difference in visual acuity. We also found significant differences between the two eyes in PWA in the strength of the correlation between visual cortical GABA and interocular suppression: for both cross- and iso-oriented surrounds, individuals with less GABA generally had weaker suppression of the fellow eye by the amblyopic eye and stronger suppression of the amblyopic eye by the fellow eye.

### Isolating perceptual suppression by controlling for baseline contrast discrimination performance

Our surround suppression task required subjects to determine which quadrant in the annulus had a higher contrast than the other three quadrants, and the contrast difference between the quadrants at threshold quantified task performance. These contrast discrimination thresholds are influenced by both the modulatory effects of the surround and intrinsic contrast discrimination ability. This represents a potential confound for the study of perceptual suppression, particularly given that PWA exhibit many perceptual deficits when viewing with their amblyopic eye (Levi, 2020).

To control for differences in contrast discrimination across participants and eyes, we measured contrast discrimination thresholds in the absence of a surround. We found a significant difference in these baseline contrast thresholds between the fellow and amblyopic eyes in PWA, as expected (Figure 8). We then quantified surround suppression by expressing contrast discrimination thresholds in the presence of a surround as multiples of the baseline threshold for that participant and eye (Figure 3). This effectively controlled for variations in intrinsic contrast discrimination ability across participants and eyes and enabled us to assess task performance with a metric that isolates the effects of the surround on contrast discrimination.

### Orientation selectivity of perceptual surround suppression

Our surround suppression task measures the effects of a high contrast surround on contrast discrimination of a center (annulus) stimulus in both monocular and dichoptic configurations. We replicated previous studies (Xing and Heeger, 2001; Yoon et al., 2009; Kosovicheva et al., 2012) showing that the magnitude of surround suppression depends on the relative orientation of the center and surround stimuli, with iso-oriented stimuli producing the strongest surround suppression (Figure 10).

### Visual cortical γ-aminobutyric acid, interocular suppression, and surround suppression

Research in animal models has suggested that intracortical GABAergic inhibition is a major contributor to surround suppression (Adesnik et al., 2012; Nassi et al., 2013), but there is also evidence for other mechanisms, including a reduction in subcortical excitatory inputs (Ozeki et al., 2004; Ozeki et al., 2009). Studies in humans have also been mixed, with some supporting intracortical GABAergic inhibition in surround suppression (Zenger-Landolt and Heeger, 2003) and others favoring withdrawal of excitation (Schallmo et al., 2018). In addition, recent studies have suggested that there are distinct neural mechanisms underlying intraocular and interocular surround suppression (Schallmo and Murray, 2016). In the present study, both intraocular (monocular) and interocular (dichoptic) stimulus configurations revealed differences between the DE and NDE in the relationship between visual cortical GABA and surround suppression in PWA.

GABAergic inhibition has also been implicated in interocular suppression of neuronal and perceptual responses. In these studies, stimuli are presented to corresponding retinal locations in the two eyes, unlike surround suppression, in which the annulus and surround were presented to adjacent but non-overlapping retinal locations, either monocularly or dichoptically. Local administration of the GABA_*A*_ receptor antagonist bicuculline strongly reduced interocular suppression of sensory responses in primary visual cortical neurons in strabismic cats (Sengpiel et al., 2006). Perceptual suppression in binocular rivalry in NSP is correlated with visual cortical GABA levels, as measured with MRS (Robertson et al., 2016), and is modulated by pharmacological alterations of GABAergic signaling (Mentch et al., 2019). Moreover, GABA visual cortical levels and administration of the GABA_*A*_ receptor agonist lorazepam are both associated with fewer perceptual switches and longer mean perceptual duration in binocular rivalry in NSP (Van Loon et al., 2013). These results, together with evidence that intracortical GABAergic inhibition contributes to surround suppression, helped to motivate the present study by suggesting that visual cortical GABA levels might be a biomarker for aspects of interocular perceptual suppression in PWA.

### Interocular perceptual suppression in amblyopia

Multiple psychophysical studies have shown a positive correlation between interocular perceptual suppression and the depth of amblyopia (Li et al., 2011; Vedamurthy et al., 2015; reviewed in Hess et al., 2014). One apparent exception is the study by Holopigian et al. (1988), which reported a negative correlation between the magnitude of interocular suppression and the depth of amblyopia. However, Holopigian et al. (1988) only studied suppression of the amblyopic eye by the fellow eye, using a small (1.2°) stimulus.

Physiologically, interocular suppression of responses in cortical area V2 is highly correlated with depth of amblyopia in strabismic macaque monkeys (Bi et al., 2011). Studies in PWA that have accounted for differences in monocular thresholds in the two eyes have found that suppression of the amblyopic eye by the fellow eye is similar to that observed in individuals with normal vision, but suppression of the fellow eye by the amblyopic eye is abnormally weak (Huang et al., 2012; Ding et al., 2013; Ding and Levi, 2014; Zhou et al., 2018; Gong et al., 2020). This pattern of asymmetric contrast gain (Ding et al., 2013; Ding and Levi, 2014) was also observed in responses to dichoptic masking stimuli in cortical areas V1 and V2 of amblyopic macaque monkeys, in which suppression of amblyopic eye responses by presentation of a mask to the fellow eye was normal, while the amblyopic eye was ineffective in suppressing responses to visual stimuli presented to the fellow eye (Shooner et al., 2017).

These studies are consistent with the idea that at least some visual deficits in amblyopia are due to insufficient suppression of fellow eye responses by inputs from the amblyopic eye. Our results support this notion by relating both the depth of amblyopia as well as the differences between the DE and NDE in surround suppression to visual cortical GABA levels in PWA.

Given the relationships between visual cortical GABA and perceptual and clinical measures of interocular interactions in amblyopia that we observed, it is perhaps surprising that there was no significant difference between PWA and NSP in overall visual cortical GABA levels in our study. It may be that correlations of GABA levels with a continuous variable (like perceptual suppression or depth of amblyopia) may be more sensitive than a categorical comparison of GABA levels in PWA and NSP. It is also possible that the abnormal visual experience that causes amblyopia alters the relationship between visual cortical GABA concentrations and perceptual outcomes, perhaps because of a compensatory process that results in the characteristic perceptual deficits observed in amblyopia.

### Limitations

Challenges in recruiting PWA that met the clinical criteria and the large number of psychophysical and MRS sessions that were required for our study imposed limits on the sample sizes for measuring correlations between visual cortical GABA levels and perceptual measures in PWA. Replicating some of the results that we report here in a larger sample with greater statistical power is an important future direction. However, evidence from three separate GABA/behavior correlations supports a relationship between reduced GABAergic visual cortical inhibition and abnormal interocular interactions in amblyopia. First, lower levels of GABA were significantly correlated with greater severity of amblyopia, as measured by interocular acuity difference. Also, less visual cortical GABA was associated with relatively weaker perceptual suppression of the fellow eye by the amblyopic eye and relatively stronger suppression of the amblyopic eye by the fellow eye. The two eyes significantly differed from each other in the correlations between visual cortical GABA and interocular suppression for both cross-oriented and iso-oriented surrounds in PWA. Even though we did not perform corrections for multiple comparisons, these converging results from three distinct perceptual data sets provide strong evidence for an important role of intracortical GABAergic inhibition in balancing interocular interactions in PWA and suggest that visual cortical GABA levels could be a biomarker for amblyopia.

### Visual cortical GABAergic inhibition as a potential therapeutic target in amblyopia

Our results support a framework in which people with severe amblyopia have reduced visual cortical GABA levels that are associated with abnormally weak suppression of the fellow eye by the amblyopic eye. Pharmacological enhancement of GABAergic signaling in visual cortex could therefore be a possible treatment for the symptoms of amblyopia in adults.

Pharmacological elevation of GABAergic inhibition could also be combined with other therapeutic interventions that influence visual cortical activity. There is substantial evidence of rapid activity-dependent regulation of visual cortical GABA in the adult visual system. Short-term (2.5 h) monocular deprivation in NSP reduces visual cortical GABA, as measured with MRS (Lunghi et al., 2015). Also, opening the eyes in darkness decreases GABA levels in visual cortex of NSP, and visual cortical GABA levels during visual processing predict performance on a visual orientation discrimination task (Kurcyus et al., 2018). In addition, several weeks of monocular deprivation in adult macaque monkeys reduces histochemical markers for GABA and glutamic acid decarboxylase (the enzyme that synthesizes GABA) specifically in deprived-eye ocular dominance columns in primary visual cortex (Hendry and Jones, 1986). Interventions that enhance GABAergic signaling could be coupled with existing behavioral training procedures that use dichoptic tasks to improve visual function in the amblyopic eye (Hess et al., 2010; Li et al., 2013; Vedamurthy et al., 2015). We note that most of these procedures have been aimed at perceptually “balancing” input from the two eyes. However, our results indicate that an alternative approach may be to train the amblyopic eye to suppress the fellow eye more effectively.

## Conclusion

In summary, we found a negative correlation between the depth of amblyopia (interocular difference in visual acuity) and GABA concentration that was specific to visual cortex and was not observed in a sensorimotor cortical control region. In addition, the two eyes of PWA differed in their relationships between dichoptic interocular suppression and visual cortical GABA levels, for both cross- and iso-oriented surrounds: visual cortical GABA levels tended to be more positively correlated with perceptual suppression of the fellow eye by the amblyopic eye and more negatively correlated with suppression of the amblyopic eye by the fellow eye. These findings indicate that therapeutic interventions to enhance the ability of the amblyopic eye to suppress the fellow eye through intracortical GABAergic inhibition may be a promising avenue of future research in the treatment of adult amblyopia.

## Data availability statement

The datasets presented in this study can be found in online repositories. The names of the repository/repositories and accession number(s) can be found below: https://github.com/smerdis/mukerji_etal_2022.

## Ethics statement

The studies involving human participants were reviewed and approved by the Committee for the Protection of Human Subjects at the University of California, Berkeley. The patients/participants provided their written informed consent to participate in this study.

## Author contributions

EY, DL, and MS designed the study. EY and KB collected the data. AM, KB, and EY analyzed the data. All authors contributed in writing the manuscript.

## Footnotes

1
https://osf.io/3gsdt/
